# Response to the Letter to Editor Regarding “Lateral Approach and Combined Lateral and Anteromedial Approach for Surgical Treatment of Terrible Triad of Elbow: A Meta-Analysis”

**DOI:** 10.30476/BEAT.2020.86026

**Published:** 2020-07

**Authors:** Mukesh Kumar Meena, Karmbeer Singh, Sanjay Meena, Chetan Kumbhare, Dushyant Chouhan

**Affiliations:** 1 *Department of Orthopedics, LHMC, New Delhi, India *

**Dear editor,**

We would like to thanks the readers of the Bulletin of Emergency And Trauma (BEAT) for their valuable comments regarding our recently published article. We appreciate their interest in our article [1]. We acknowledge the heterogonous nature of studies included in the analysis and this was duly mentioned in the manuscript. This is one of the limitations of the present meta-analysis. The readers rightly pointed that a discrepancy in the conclusion and forest plot diagrammatic representation. We take this opportunity to correct it. The correct statement in result and conclusion should be as follows:

“There was no difference in Mayo elbow performance score (MEPS) between LA and CML groups (MD: -3.31, 95% CI: -7.23 to 0.62, p=0.10)”.

Another concern by the reader was the exclusion of two studies from the meta-analysis [2, 3]. We did not include the two studies mentioned by the readers because of the following reasons.

1. The study by Pierrart *et al*., [2] was not included since it was not a comparative study and one of the selection criteria of the meta-analysis was studies which compared lateral to combined lateral and anteromedial approach for surgical treatment of terrible triad of elbow.

2. The study by Liu *et al*., [3] was not included since all the patients in that study were operated by combined approach. I am not sure, how the reader analyzed the data after adding this study, since there was no comparative group. 

We assume that further high quality randomized controlled trials (RCTs) are required to support our conclusion.

## Conflict of Interest:

None declared.
